# The Effect of Fulvic Acids Derived from Different Materials on Changing Properties of Albic Black Soil in the Northeast Plain of China

**DOI:** 10.3390/molecules24081535

**Published:** 2019-04-18

**Authors:** Mahendar Kumar Sootahar, Xibai Zeng, Shiming Su, Yanan Wang, Lingyu Bai, Yang Zhang, Tao Li, Xiaojia Zhang

**Affiliations:** 1 Institute of Environment and Sustainable Development in Agriculture, Chinese Academy of Agriculture Sciences, Beijing 100081, China; mahender_935@yahoo.com (M.K.S.); sushiming@caas.cn (S.S.); wangyanan@caas.cn (Y.W.); bailingyu@caas.cn (L.B.); zhy2198@163.com (Y.Z.); tllcu@caas.com (T.L.); zhangxiaojia871230@163.com (X.Z.); 2 Department of Soil Science, Sindh Agriculture University, Tando Jam 70060, Pakistan

**Keywords:** fulvic acid, soil fertility, soil organic carbon fractions, albic black soil

## Abstract

Despite low fertility and content of organic carbon in albic black soil, grains are grown in this type of soil in the northeast plain of China in order to find ways to improve the soil’s fertility and crop production. We carried out pot experiments of maize applied with one of three different treatments of fulvic acids (FA) derived from different parent materials: Plant-derived solid (PDSF), mineral-derived liquid (MDLF), and plant-derived liquid (PDLF) applied at respective rates of 2.5, 5, and 5 g kg^−1^ as well as a control applied at 0 g kg^−1^. The results showed that soil organic carbon and light fraction C was greater by 29% to 21% and 38% to 21%, respectively, among the treatments compared to that of the control. Similarly, available N content was significantly greater in the PDLF treatment, and P content was also significantly greater in the PDSF treatment. In contrast, available K and extractable Mg contents were lower, as well as organic–inorganic degree complexes and organic–inorganic composites in the PDSF, MDLF, and PDLF treatments compared with those of the control. Further results showed that MDLF and PDLF Fulvic acids (FA) accelerated plant growth, while PDSF limited plant growth. Our study provides empirical evidence that addition of fulvic acid from MDLF and PDLF had more positive effects on soil properties and plant growth than fulvic acid from PDSF. This investigation suggests that application of fulvic acid in liquid form can improve nutrient availability and affect other important chemical, biological, and physical properties of soils.

## 1. Introduction

In recent years, many advanced techniques have been adopted towards the improvement of the quality and quantity of agricultural production. The new developments in agriculture depend not only on mechanization and new hybrid seeds, but also on soil properties improvement to increase crop productivity and improve land use management. However, inappropriate soil management can lead to food scarcity and low productivity because of the depletion of beneficial soil elements to crops. Albic black Luvisols (Argillic) soil, an important soil type for grain production, covers 5.96 million hectares in China and is widely distributed in many countries [1,2]. The soil is a clay loam (Hapludoll, based on soil survey staff 1990) and the main soil-forming parent material is clay sediments. The black soil is an Udic Isohumisols, a suborder of the Isohumisols order according to the soil taxonomy of China [3]. The soil is composed of vermiculite and illite minerals with a high cation exchange capacity (CEC) [4]. Soil aggregate formation occurs in this soil by the accumulation of organic carbon and formation of organic–inorganic complexes [5]. Albic soil has been classified as a low-yield soil because organic matter content rapidly decreases due to a thin layer of humus, where wetting and drying cycles lead to the alternation of the oxidation-reduction process that bleaches the sub-surface of soil [1]. Strong acidity and poor permeability are the main reasons for low fertility [5]. 

To reduce the oxidation-reduction issue and increase soil fertility, many scientists began to apply humic substances such as fulvic and humic acids to improve soil properties and plant growth. Crop production on Albic black soil is often limited by the unavailability of nutrients. However, crop yields on this type of soil differ substantially between farms, which may be due to differences in fertilization history and organic substance accumulation [1]. In general, the application of organic substances (Fulvic/Humic) is essential to achieve good yields on this type of soil. These organic substances are made up of different nitrogenous complexes comprising decayed amino and aromatic complexes [6]. In the presence of carboxyl (COOH^−^) and phenolic (OH^−^) groups, these organic complexes affect soil properties and physiological properties of plants [7]. It has been stated earlier that fulvic acids (FA) have direct and indirect involvement on plant growth [8,9]. Soil aggregation, aeration, microbial growth, organic matter mineralization, water holding capacity, and transport of macro and micronutrients are improved by humic substances indirectly [8,9,10]. Cell walls, photosynthesis, and the respiration rate in plants are directly affected by humic substances [11]. Overall, it seems that plant physiological characters are directly affected by fulvic acids (FA), which is primarily known to enhance root growth and nutrient uptake [12]. Due to the above facts, fulvic acids (FA) was used in this investigation on whether it would increase the production of crops and the availability of essential nutrients in Albic black soil. Fulvic acids (FA) derived from different parent materials were applied to the soil to increase the production of a maize crop.

The aim of our work was to determine how fulvic acids (FA) can affect soil properties and plant growth parameters in maize grown in Albic black soil. We hypothesized that fulvic acids (FA) improves crop production by improving soil physical and chemical properties that increase soil organic matter content and reduce the issues regarding low soil fertility. Fulvic acids (FA) were derived from three different parent materials and used as treatments—plant-derived solid (PDSF), mineral-derived liquid (MDLF), and plant-derived liquid (PDLF) fulvic acids (FA)—and applied at different concentrations on maize-grown soil to determine responses of various plant growth parameters (plant height, biomass, and grain weight) and soil available nutrients such as soil organic carbon (SOC), N, P, K, Ca, and Mg. The objectives of this study were to assess the effect of fulvic acids (FA) derived from different parent materials on soil chemical properties, soil organic matter fractions, soil available nutrients, and plant growth of maize grown in Albic black soil.

## 2. Results

### 2.1. Effect of Fulvic Acids (FA) on Soil Organic Carbon and its Labile Fractions

**Figure 1:**
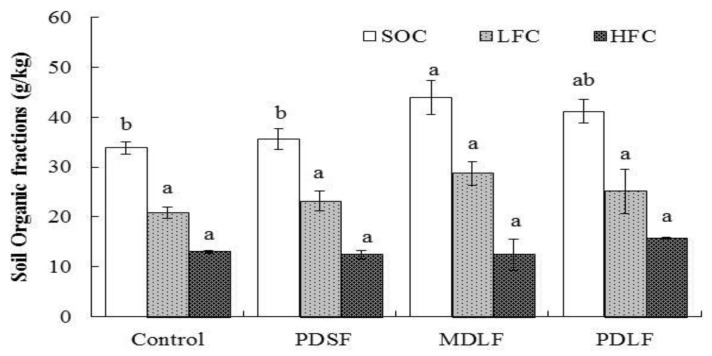
Effect of Plant-derived solid (PDSF), mineral-derived liquid (MDLF), and plant-derived liquid (PDLF) fulvic acids (FA) on soil organic carbon, light fraction C, and heavy fraction C. Error bars represent standard errors of the means (*n* = 4). The least significant differences (LSD_0.05_) are at 5% level of significance. Different letter on top of each bar showing significant differences between treatments.

The different treatments of fulvic acids (FA) significantly affected soil organic carbon content and insignificantly affected light and heavy fractions C (Figure 1). The results showed that soil organic carbon contents were greater than that of the control by 5%, 29%, and 21% in PDSF, MDLF, and PDLF. The contents of light fraction C were greater by 11%, 38%, and 21% for the same respective treatments. Heavy fraction C was greater by 21% in PDLF, but it was lower by 4.6% and 4.4% in PDSF and MDLF treatments, respectively, as compared with that of the control.

### 2.2. Effect of Fulvic Acids (FA) on Soil Chemical Properties

**Figure 2:**
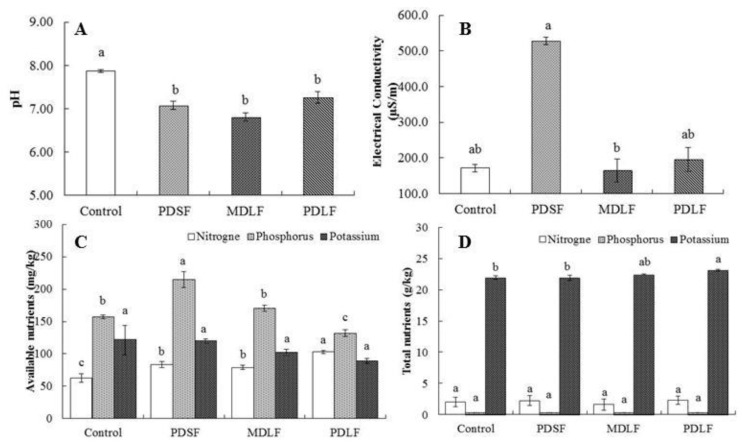
The effect of PDSF (plant-derived Solid), MDLF (mineral-derived liquid), and PDLF (plant-derived liquid) fulvic acids (FA) on (**A**) soil pH, (**B**) electrical conductivity, (**C**) soil available nutrients, and (**D**) soil total nutrients of dry soil from pot experiment. Error bars represent standard errors of the means (*n* = 4). The least significant differences (LSD_0.05_) are at 5% level of significance, different letter on top of each bar showing a significant difference between treatments.

The effect of fulvic acids (FA) was significant on soil EC and pH (Figure 2A,B), as well as on available nitrogen, phosphorus, and total potassium contents. However, effects on available potassium and total N and P were not significant among the treatments (Figure 2C,D). The results showed that soil pH was lower by 10%, 13%, and 8% in PDSF, MDLF, and PDLF, respectively, as compared with pH of the control. However, EC accumulation as greater by 208% and 14% in PDSF and PDLF, respectively, and decreased by 4% in MDLF compared to EC of the control. Similarly, available nitrogen content was lower by 32%, 25%, and 63% in the respective PDSF, MDLF, and PDLF treatments. Conversely, in comparison to the control, available phosphorus content was greater by 37% and 8.4% in PDSF and MDLF, respectively, but was lower by 16% in PDLF. Although available potassium was lower by 2%, 16%, and 26% in PDSF, MDLF, and PDLF, respectively, compared to that of the control. Similarly, total N, P, and K contents in PDSF, MDLF, and PDLF treatments were greater by between 1.95% and 13.4% than those of the control. In contrast, total N and K was lower by 19.4% and 0.22% in MDLF and PDSF, respectively, compared with those of the control.

### 2.3. Effect of Fulvic Acids (FA) on Soil Exchangeable Ca and Mg, Organic–Inorganic Compound, and Organic–Inorganic Complexes

Fulvic acid application had a significant effect on exchangeable calcium, organic–inorganic composites of SOM, and organic–inorganic compounds, and it had a non-significant effect on exchangeable magnesium (Table 1). The results showed that exchangeable calcium was greater by 3.4% in MDLF treatments but lower by 5.7% and 23.5% in PDSF and PDLF treatments, respectively, when compared with that of the control. Organic–inorganic compound levels lower by 11%, 17.8%, and 18% and organic–inorganic composite levels were lower by 0.06%, 0.09%, and 0% than that of the control in PDSF, MDLF, and PDLF treatments, respectively. Exchangeable magnesium was lower by 12%, 21%, and 3% in PDSF, MDLF, and PDLF treatments as compared with that of the control. The significant interactions of organic–inorganic compounds and organic–inorganic composites were negatively correlated with SOC, LFC, and available P and K. Similarly, Ca was negatively correlated with Mg content.

### 2.4. Effect of Fulvic Acids (FA) on Plant Growth Parameters

**Figure 3:**
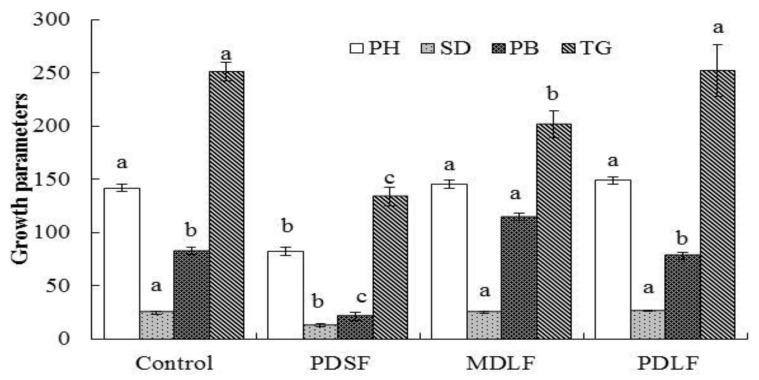
Effect of PDSF, MDLF, and PDLF fulvic acids (FA) on plant height (PH), stem diameter (SD), plant biomass (PB), and thousand-grain weight (TG) of the Maize crop; error bar shows the standard error of means (*n* = 4). The least significant differences (LSD_0.05_) are at 5% level of significance, different letter on top of each bar showing significant differences between treatments.

Plant growth parameters were significantly and insignificantly affected among the treatments (Figure 3). Results shows that plant height was respectively greater by 2.6% and 5.2% in MDLF and PDLF treatments. In contrast, plant height was lower by 42% in PDSF. Stem diameter was greater by 5.6% in the PLDF treatment and lower by 48% in the PDSF treatment as compared with diameter of the control. Plant biomass was greater by 38.5% in the MDLF treatment and was lower by 74% and 5.32% in the PDSF and PDLF treatments, respectively. The thousand-grain weight was greater by 0.4% in the PDLF treatment and was lower by 46% and 19% in the respective PDSF and MDLF treatments as compared with that of the control.

### 2.5. The Response of Fulvic Acids (FA) on Nutrient Uptake

**Figure 4:**
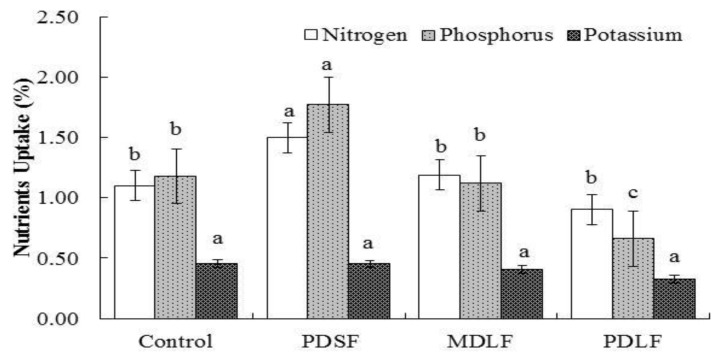
Effect of PDSF, MDLF, and PDLF fulvic acids (FA) on plant nutrients uptake of the maize crop; error bar shows the standard error of means (*n* = 4). The least significant differences (LSD_0.05_) are at 5% level of significance, different letter on top of each bar showing significant differences between treatments.

Nutrient uptake by plants was significantly affected by the fulvic acids (FA) treatments (Figure 4). The results showed that nitrogen uptake was respectively greater by 36% and 8% in PDSF and MDLF treatments; however, it was lower by 18% in the PDLF treatment. Similarly, phosphorus uptake was greater by 50% in the PDSF treatments and lower by 44% in the PDLF treatment. On other hand, potassium uptake was lower by 2%, 10%, and 28% in PDSF, MDLF, and PDLF treatments, respectively, as compared with that of the control.

## 3. Discussion

### 3.1. Effect of Fulvic Acid on Soil Organic Carbon and Organic–Inorganic Compounds

Previous studies suggest that labile pools of SOM in temperate soils are more complex to cropping practices than total soil organic C pools [13]. The light fraction C is a short-term pool of plant nutrients and is the primary fraction of soil carbon formation [14]. Due to rapid changes in C supply, LFC is considered to be an early indicator of soil quality changes [15]. However, heavy fraction C is more stable in the high-density organic-mineral fraction and has low C concentrations [16,17]. In contrast with LFC, HFC contains more processed SOM [18,19]. It can be a major sink in soil fertility because it has mineralizable C [20,21]. In our study, application of fulvic acids (FA) significantly increased soil organic carbon by 35% to 40% and light fraction C (LFC) by 23% to 29%, but it decreased C in the heavy fraction C (HFC) by about 12% of total organic C content (Figure 1). The soil organic carbon was significantly increased at MDLF and PDFL treatments, however, LFC and HFC was observed non-significant among the treatments. The increases in SOC and LFC among the treatments caused by application of fulvic acids (FA) may be due to the fact that fulvic acids (FA) are a mixture of organic particles that enhance soil organic matter content. These organic particles also improved organic matter mineralization within the soil. We observed that organic particles mobilized SOM when we mixed soil samples with NaI by sonication during the densitmatric separation process and during water rinses, which may explain the decrease of HFC. Soil OM was solubilized by a strong effect of the solid particles, minerals, and organic substances (humic and fulvic acid), during the NaI treatment [22]. These results are in accordance with the results of Crow et al. [23]; these researchers incubated forest soils for a duration of one year, and showed bulk soil have ~40% of higher recombined density fractions C than the LFC; moreover, LFC did not often decompose more C than HFC. von Lützow et al. [24] investigated soil labile and recalcitrant C in a six-year study conducting treatments of precipitation that stimulated LFC, LFC:HFC ratio, and artificial warming that reduced soil labile C (LFC and HFC). Dissimilarly, Gong et al. [25] reported that HFC and LFC were found significant after application of organic manure in conjunction with chemical fertilizers.

The organic–inorganic composite is the structural basis of soil fertility and organic–inorganic degree complexes are an important indicator in evaluating soil fertility [21]. The results obtained in our experiment show significantly lower amounts of both organic fractions among the treatments when compared with those of the control (Table 1). Lower amounts of organic–inorganic composite and organic–inorganic degree complexes may be caused by the different sources of fulvic acids (FA) and the different fulvic acids (FA) concentrations applied. The different parent material suggests that functional groups of fulvic acids (FA) are differ from each other and may cause the loss of important soil fertility indicator.

### 3.2. Effect of Fulvic Acids (FA) on Soil Fertility

Soil EC and pH are also important gauges of soil fertility. They have direct effects on buffering capacity, which determines the rate of change in pH during soil acidification and liberates carbon dioxide [26]. Acidification causes fulvic acids (FA) to remain in soil solution; fulvic acids (FA) is soluble in water under all pH conditions [27]. Our study showed that fulvic acids (FA) significantly decreased the soil pH (Figure 2A). The acidity may have been the result of quinone groups such as carboxyl, phenolic, and hydroxyl groups that easily lower the soil pH [8]. Fulvic acid also influences the growth of soil microbial biomass and microbial activity. fulvic acids (FA) are the active ingredients and provides carbon and energy to microorganisms. Fulvic acids (FA) have COOH and OH groups and phenolic groups, which have ability to inprove soil structure and fertility [28]. Similarly, fulvic acids (FA) have a direct impact on the soil physiochemical properties, CEC, and stabilization of soil structure, along with mineral nutrients [28]. It strongly affects the release of macronutrients, N, P, and K [8,9]. Our results indicate that fulvic acids (FA) significantly increased N content in soil as compare with control; higher N content was observed at PDLF (Figure 2C,D). These results are similar with the results of References [8,29], who reported that humic acid (HA)/fulvic acids (FA) application increases the NPK content of soil. However, Singaram et al. [30] stated that soil total N content after the application of HA at a rate of 20 kg ha^−1^ was increased by 28% and 29%. The presence of 7% N in lignite coal is probably the reason for the increase in total N content in soil [8]. Inhibition of urease activity by HA led to reduced N losses, thereby increasing the N concentration in the soil. Similarly, available P in soils was significant increased at PDSF and decreased by PDLF fulvic acids (FA), however Total P content was found to be non-significant between treatments (Figure 2C,D). Phosphorus fixation was reduced by fulvic acid and increased the solubility of P concentration in soil [31]. These findings are related to those reported by Han et al. [32], as they suggested that humic acid have the ability enhance the uptake of phosphorus in plants. However, Du et al. [33] stated that the addition of HA to mono-calcium phosphate resulted in increased concentrations of water-extracted P, acid-extracted P, and Olsen P, which was not supported by the lower amount of soil-available K observed in this study (Figure 2C,D). Possibly, the fulvic acids (FA) concentration we applied was not high enough to cause the release of fixed K in the soil. However, Liu et al. [34] reported that humic substances increasing the potassium fixation in soil by dissolving k-bearing minerals.

According to many researchers, humic substances (fulvic, humic, and humin) may enhance plant growth and nutrient uptake and diminish the uptake of hazardous elements and improve plant metabolism [11]. However, there is a dearth of research on fulvic acids derived from different parent material and its effects on soil organic matter fractions, soil-available nutrients, and plant growth parameters. Reference [8] stated that the application of lignitic coal HA increased plant growth and nutrient uptake and these responses are mainly associated with the potential of HA to improve the biochemical environment of the soil. The present study indicated that application of MDLF and PDLF fulvic acids (FA) increased plant growth and nutrient contents in the soil. These results support Khan et al.’s [35] findings that coal-derived humic acid significantly increased the physical properties of sandy and clayey loam soils by enhancing soil retention of nutrients and consequently increasing plant growth. Saruhan et al. [9] also suggested that application of fulvic acids (FA) enhances plant growth parameters as well as uptake of mineral elements in a maize crop.

Besides primary macronutrients (N, P, and K), the secondary macronutrients calcium and magnesium also play a significant role in the soil–plant relationship; these elements are vital for plant growth and, if present in adequate amounts in the soil, improve the fertility. However, our results indicated that fulvic acids (FA) decreased calcium and magnesium contents in soil (Table 1). It is possible that the fulvic acids (FA) were saturated with unwanted cations and important metal contaminants that may have lowered calcium and magnesium contents [27]. On the other hand, Khaled et al. [36] reported that foliar application of fulvic acids (FA) had a non-significant effect on calcium and magnesium contents compared to their control. Fulvic acids (FA) break up compacted soils which allows for greater water penetration and root growth and development in clayey soils; however, in sandy soil, fulvic acids (FA) enhanced the organic material which increased the water retention, improved root growth, and retained or leached vital plant nutrients in sandy soil [36].

### 3.3. Effect of Fulvic Acids (FA) on Plant Growth

The increase in plant growth, stem diameter, plant biomass, and thousand-grain weight in MDLF and PDLF fulvic acids (FA) treatments confirms that the addition of fulvic acids (FA) has direct influence on plant growth parameters (Figure 3). Reductions in growth parameters due to PDSF may be caused by the application of a low concentration of PDSF fulvic acids (FA). Another reason may be that the soil-applied fulvic acids (FA) did not completely dissolve in the soil as liquid fulvic acids (FA) would have easily dissolved in soil. The liquid or foliar form of fulvic acids (FA) are more effective for plant growth and metabolic sites in plant cells because they contain many small microbes, which polarized the soil and available nutrients to plants [7]. These results are in contrast to the findings of Reference [36], who reported that soil-applied HA and foliar application of HA increased plant growth of wheat. Reference [8] suggested that application of lower concentrations (50 to 100 mg kg^−1^) in soil was effective in stimulating the yield of maize as compared with the control, and greater level of 150 to 300 mg kg^−1^ produced no significant effect on maize yield. The results in yield and yield components that were observed in this study support previous findings [29]. Similarly, Delfine et al. [37] reported approximately 23–26% increases in wheat grain yield due to HA applications. The results concluded in this study that application of liquid or foliar forms of fulvic acids (FA) caused increased contents in plant growth parameters, which support the results of Çelik et al. [38]. They concluded that foliar spray of 0.1% and 0.2% HA increased the yield of maize by 14% and 13%, respectively. The increase in yield and yield component characters of maize could be attributed to direct or indirect effects of fulvic acids (FA) on plant growth and development. Fulvic acid may stimulate root growth and affect root function to exude organic acids that leads to increased nutrient uptake and consequently, improved growth and yield of crops [39].

## 4. Material and Methods

### 4.1. Experimental Soils and Fulvic Acids (FA)

Soil samples were allocated at the depth of 0–20 cm from field of Qiqihar city; district Jianhua (47°21′ N, 123°55′ E) located in west part of Heilongjiang province northeast of China. The soil was previously cultivated with maize crop with an area of approximately 584 forms. The experiment was conducted at the Institute of Environment and Sustainable Development (IEDA) experimental site (40°09′ N, 116°92′ E). The sampling location is within the subtropical windward climate zone with an average annual rainfall of 265 mm and an average temperature of 44 °C [1]. Physical and chemical properties were determined: Soil particle size analysis was determined using a Master sizer 2000E (Malvern, UK) laser diffract meter [40]. pH was measured potentiometrically in a 1:2.5 soil water extract (DDS-307 EC meter, Shanghai Bante Instrument CO., Ltd., China) and pH meter (Mettler Toledo 320-S. Shanghai Bante Instrument Co., Ltd., Shanghai, China). Soil organic carbon (SOC) was determined following wet digestion with sulfuric acid (H_2_SO_4_) and potassium dichromate (K_2_CrO_7_) (Walkley and black 1934). Total N, P, and K contents of plant shoots and leaves of maize were analyzed calorimetrically after digestion with (H_2_SO_4_ + HCLO_4_), briefly described by Parkinson et al. [41]. Soil-available P was measured using 0.5 M NaHCO_3_ (pH 8.5) extract followed by a visible light spectroscopic analysis of a blue colored complex according to the method in (UV-VIS spectrophotometer, Model UV-1208, Shimadzu, Kiyoto, Japan) Reference [42]. Available K was determined using 1 N ammonium Acetate (NH_4_OA_C_) extraction followed by emission spectroscopy (FP 6410, Shanghai Bante Instrument Co, Ltd., Shanghai, China) [43]. Some soil physiochemical properties examined in this exploration are shown in Table 2. Silty clay loam soil was used in this experiment, which is acidic in pH, low in lime (CaCO_3_), soil organic matter, and phosphorus contents, however, nitrogen and potassium content were in adequate range. The soil was not adequate for obtaining high yields in crop production.

### 4.2. Experimental Design

The present study used a completely randomized design that included four concentrations of fulvic acids (FA); 0 for the control and 2.5, 5, and 5 g kg^−1^ for the PDSF, MDLF, and PDLF treatments, respectively, in an area of 50 × 60 cm plot. There were three replications for each treatment. The soil-applied fulvic acids (FA) was obtained from Shandong Quan Linjia fertilizer Co. Ltd (Shandong, China). Chemical composition of the three fulvic acids (FA) are presented in Table 3. The initial material of fulvic acids (FA) was derived by the International humic acid society (IHSS) method. Air-dried soil was passed through a 5-mm sieve. Solid and liquid applications of fulvic acids (FA) were as follows: PDSF fulvic acids (FA) were mixed with soil up to a total of 15 kg in a large bowl and then put in plastic pots, while MDLF and PDLF fulvic acids (FA) were applied with water into pots with 15 kg of soil. The pots were 27 and 29.5 cm in diameter and height, respectively. The pots were buried in the field so that the tops of the pots were level with the soil surface. Five maize seedlings were planted in each pot. Compound fertilizer with a composition of 25% N, 14% P_2_O_5_, and 7% K_2_O, was applied three separate times to a total of 15 g pot^−1^ (5 g at sowing, 5 g after transplanting, and 5 g at the maturation stage). All pots were irrigated equally after every two or four days during the experiment. Plants were harvested at the mature stage and dried at 65 °C. Each harvested plant part such as leaves, stems, and grains were separated according to the treatments and labeled carefully for the assessment of agronomic parameters of plant height, stem diameter, total plant biomass, and thousand-grain weight.

### 4.3. Organic–Inorganic Compound and Organic–Inorganic Composites Analysis

In this study, we separated soil labile and recalcitrant fractions of soil organic matter (SOM) by density fractionation, which is one of the physical fractionation methods used widely. The light fractions of low density (<1.7 g cm^−3^) are partly decayed plant and animal products, while heavy fractions of high density (>1.7 g cm^−3^) consist of a humic substance that is generally mineral associated [44,45]. Specifically, a sample of ~5 g was put into a well-sterilized centrifuged tube added by 25 mL of NaI solution with a density around 1.7 g cm^3^. A mechanical shaker was used to shake centrifuged tubes for 30 mins; after, the tubes were transferred to centrifuge at 3000 rpm for 10 mins. Light fraction soil that was moving on the surface was filtered using fiberglass filter in a Buchner funnel. We repeated the above process 2 to 3 times to separate light and heavy fractions. Remaining material was washed by adding 25 mL deionized water in a centrifuge machine about three times. The light fraction was washed with 25 mL of 0.01 M CaCl_2_ and then 25 mL of deionized water. Both the light and heavy fractions were dried at 60 °C for 48 h, weighed and ground to determine C content, after which both fractions of organic and inorganic complexes were calculated by the following equation:

Organic–inorganic compound % = HC × HW/SW × SC × 100(1)

Organic–inorganic composite g/kg = HC × HW/SW(2)

where HC (g/kg) is the carbon content in the heavy fraction; HW (g/kg) is tube weight subtracting the final weight of soil after removal of light and heavy fraction; SW (g) weight of the air-dried soil; and SOC (g/kg) is the content of soil organic carbon.

### 4.4. Calculation and Statistical Analysis

Data are expressed as means ± SE (standard error). Means (*n* = 3) were subjected to one-way analysis of variance (ANOVA) and compared using Bonferroni multiple comparison tests at the significant level of *p* < 0.05. Statistical analyses were performed using the software developed by Statistics Product and Service Solution (SPSS, Version 21.0, Chicago, IL, USA).

## 5. Conclusions

Fulvic acid is an essential part of soil organic structure. It increases cell division rates and promotes greater root development and consequently causes the growth of stronger plants that are likely more resistant to plant diseases (Khaled and Fawy 2011). Fulvic acid has been used by many scientists, agronomists, and farmers to improve soil physical properties and increase exchange capacity and buffering qualities of soils. Due to decline of organic carbon and presence of organic mineral complex, albic luvisols soil has become low in available nutrients and crop production. To evaluate soil fertility, increase the production, and break up the organic mineral complex, soil physical, chemical properties, and plant growth characteristics were determined. Our results show that application of fulvic acids (FA) increased soil physical and chemical properties and also increased growth parameters. Besides increasing production, fulvic acids (FA) increased nutrient uptake. It was also observed that application of MDLF and PDLF fulvic acids (FA) increased soil organic carbon and light fraction C contents. Application of these two types of fulvic acids (FA) also enhanced plant height, biomass, plant stem diameter, and thousand-grain weights, whereas application of PDSF fulvic acids (FA) decreased these responses. We conclude that MDLF and PDLF application enhances soil properties and plant growth parameters of maize. Overall, we found that fulvic acids (FA) application in liquid forms performs better than the solid fulvic acids (FA) in Albic black soil. Economic levels of application should be determined to improve soil management strategies and should not exceed 5 g kg^−1^ in the soil.

## Figures and Tables

**Table 1:** Influence of fulvic acids (FA) on exchangeable calcium, magnesium, organic–inorganic degree compound and organic–inorganic composite after maize harvested (mean ± standard error; *n* = 4).

Treatments g/kg	Ca meq/L	Mg meq/L	Organic–Inorganic Degree Compound (%)	Organic–Inorganic Composite (g/kg)
Control	240.1 ± 5.10 ^a^	26.7 ± 4.34 ^a^	32.55 ± 1.32 ^a^	1.09 ± 0.02 ^a^
PDSF	226.4 ± 22.6 ^a,b^	23.4 ± 0.25 ^a^	28.84 ± 1.24 ^a,b^	1.02 ± 0.01 ^b^
MDLF	248.3 ± 6.43 ^a^	21.1 ± 0.70 ^a^	26.77 ± 1.26 ^b^	1.08 ± 0.09 ^a^
PDLF	189.5 ± 17.25 ^b^	25.9 ± 0.83 ^a^	26.74 ± 1.40 ^b^	1.09 ± 0.01 ^a^

Data are mean of (*n* = 4). Means followed by different letters (a, b) are significantly different from each other at (*p* < 0.05). Values are meant ± standard error, nd 3. Heavy fraction carbon (HFC), light fraction carbon (LFC), PDSF plant-derived (Solid), mineral-derived (Liquid), and plant-derived (liquid) fulvic acids (FA).

**Table 2:** Physicochemical properties of soil used in the experiment.

Items	Value
Electrical conductivity µS/cm	0.31
pH	7.88
Soil Organic Carbon (g/kg)	8.44
Cation Exchange Capacity	21.6
Available Nitrogen (mg/kg)	66.0
Available Phosphorus (mg/kg)	0.44
Available Potassium (mg/kg)	78.0
Total Nitrogen (g/kg)	0.70
Total Phosphorus (g/kg)	0.39
Total Potassium (g/kg)	19.3
2~0.2 mm	10.89
0.2~0.02 mm	33.46
0.02~0.002 mm	28.69
>0.002 mm	26.95
Texture Class	Silty Clay Loam

**Table 3:** Elemental composition of plant-derived liquid, mineral-derived liquid, and plant-derived solid fulvic acid.

FA Type	N	C	H	S
	%			
**PDSF**	5.39	25.31	5.75	8.47
**MDLF**	10.29	52.476	9.74	14.84
**PDLF**	10.78	50.61	11.56	16.96

PDSF: Plant-derived solid, MDLF: Mineral-derived liquid, and PDLF: Plant-derived liquid.
